# Potential Use of *Annona* Genus Plants Leaf Extracts to Produce Bioactive Transdermal Patches by Supercritical Solvent Impregnation

**DOI:** 10.3390/antiox10081196

**Published:** 2021-07-27

**Authors:** María Teresa Fernández Ponce, Cristina Cejudo Bastante, Lourdes Casas Cardoso, Casimiro Mantell, Enrique J. Martínez de la Ossa, Clara Pereyra

**Affiliations:** Chemical Engineering and Food Technology Department, Wine and Agrifood Research Institute (IVAGRO), University of Cadiz, 11510 Cadiz, Spain; cristina.cejudo@uca.es (C.C.B.); lourdes.casas@uca.es (L.C.C.); casimiro.mantell@uca.es (C.M.); enrique.martinezdelaossa@uca.es (E.J.M.d.l.O.); clara.pereyra@uca.es (C.P.)

**Keywords:** *Annona*, phenolic compounds, biopolymers, supercritical impregnation, wound dressings, antioxidant capacity, antimicrobial capacity

## Abstract

The objective of the present work was to develop a bioactive transdermal patch functionalized with *Annona* leaf extracts (ALE) by means of supercritical impregnation technique. The potential of six different *Annona* leaf extracts (ALE) obtained with the enhanced solvent system formed by carbon dioxide + ethanol/acetone was evaluated taking into account the antioxidant activity, total phenol composition and global extraction yields. For the impregnation of ALE, two drug supporting systems were tested: hydrocolloid sodium carboxymethyl cellulose (NaCMC) and polyester dressings (PD). The effect of the impregnation conditions, including pressure (*P*), temperature (*T*), percent of co-solvent (ethanol) and ALE/polymer mass ratio, was determined with regard to the loading and the functional activity of the impregnated samples. The optimal impregnation conditions of ALE were established at 55 °C and 300 bar which led to obtained transdermal patches with antioxidant and antimicrobial capacity. In order to understand the behavior of the process, the homogeneity of the samples in the vessels was also evaluated. The best results were obtained at higher proportions of co-solvent in the system.

## 1. Introduction

It is a fact that around 74% of the current drugs in the market are administered orally, which is a route that does not seem to be as effective as desired. Transdermal drug delivery systems (TDDS) in the form of transdermal patches have emerged as an alternative novel drug administration route [1]. TDDS have the capacity to directly deliver drug/active compounds through the patient’s skin into his/her circulatory system, thus avoiding the digestive system, were the drug molecules can be inactivated by hepatic metabolism. This administration route represents an improvement in drug bioavailability, which in turn allows a reduction of drug doses while keeping plasma levels steady over extended periods of time. Moreover, transdermal patches are not only a non-invasive and painless administration form, but they also avoid or minimize a number of harmful side effects such as gastric irritation, diarrhea or nausea. Therefore, transdermal patches enhance drugs’ therapeutic efficiency and encourage patients to follow through their whole treatment [1].

Transdermal patches have been developed for a diversity of applications, including postmenstrual syndrome, pain relief, hypertension, diabetes, Alzheimer or smoking cessation [2,3]. The administration device is usually formed by a polymeric matrix, the drug/active ingredient and, in some cases, a permeation enhancer. The polymeric matrix controls the release of the drug and it should be chemically non-reactive, non-toxic and inexpensive. Natural polymers, such as cellulose derivatives, gelatin, waxes, gums and chitosan, are often used because of their biodegradability and biocompatibility [2].

Bioactive compounds of natural origin have also gained considerable attention as active ingredients. In this sense, *Annonaceae* is a botanical family that comprises about 130 genera and 2.500 species with interesting biomedical applications [4]. In particular, *Annona* genus (*Annonaceae* family) includes more than 100 species from tropical and subtropical regions around the world [5]. Five areas worldwide areas are characterized by a Mediterranean climate, including the European Mediterranean Basin, a predominant area for cherimoya cultivation [5]. *Annona* genus had already attracted the attention of the scientific community in the past decades because of their wide variety of biomedical properties, including anti-inflammatory, antioxidant, bacteriostatic and cytotoxic activity. Different bioactive compounds including flavonoids, polyphenols, terpenoids, alkaloids and acetogenins have been isolated from *Annona* species [6,7,8].

According to the literature, conventional organic solvent extraction methods have achieved low recoveries of bioactive compounds from *Annonaceae* plants. This may be because some of the active substances in the *Annona* genus, like acetogenins present a low-polarity and they are also altered by high temperatures. Yang et al. [9] explored and confirmed the efficiency of supercritical CO_2_ to produce *Annona* seed extracts. Nevertheless, although supercritical CO_2_ (scCO_2_) is a non-polar solvent, other polar modifiers or co-solvents, such as ethanol, are required to recover the high polarity compounds that are present in *Annona* plants, such as flavonoids, tannins, alkaloids or phenolic compounds [4,10]. When the percentage of co-solvent is over 10%, the process is commonly known as enhanced solvent extraction (ESE). This extraction technique requires a smaller amount of liquid organic solvents compared to other conventional extraction techniques. CO_2_ is a green inert solvent that reduces oxidation reactions and prevents the degradation of the active substances when added to the mobile phase in greater proportions. This addition of CO_2_ to the solvent mixture also favors matter transfer phenomena by reducing the solvent surface tension, which in turn enhances the penetrability of the solvent into the matrices. CO_2_ also enables an increment of the operating temperature, which improves solubility and analyte diffusion rate [11].

Despite their interest and biomedical properties, as far as the authors know *Annona* bioactive compounds have not been used in medical devices for the controlled delivery of drugs. Supercritical solvent impregnation (SSI) is one of the most innovative techniques that have been used to functionalize polymers. scCO_2_ can, in fact, be used to impregnate active compounds into polymeric matrices, with some advantages with respect to other conventional techniques, including the preservation of thermolabile compounds, and an easier penetration of the active compounds into the polymeric matrix thanks to the plasticizing effect of the scCO_2_ [12]. The results that have been obtained to date with regard to the production of bioactive patches are rather promising [13,14,15]. For instance, Pires et al. [16] achieved greater loadings of thymol and beta-carotene into chithosan-alginated wound dressings by SSI than by conventional methods. Da Silva et al. [14] evaluated the effectiveness of SSI to impregnate a natural-based commercial wound dressing (Promogran^®^) with a spilanthol-enriched extract obtained from jambu (*Spilanthes acmella*) flowers. Pascoal et al. [17] proposed SSI for the production of biopolymer wound dressings loaded with copaibal oil for a cutaneous treatment against leishmania, and Dias et al. [18] used SSI to impregnate N-carboxybutyl chitosan (CBC), collagen/cellulose (Promogran^®^) and hyaluronic acid-based (Hyalofill^®^) polymeric matrices/dressings with an extract obtained from jucá (*Libidibia ferrea*).

Other natural-based wound dressings such as hydrocolloid sodium carboxymethyl cellulose (NaCMC) seem to hold a considerable interest regarding functionalization processes, since wound dressings based on CMC are generally regarded as nontoxic, non-irritant, and biocompatible. More specifically, NaCMC, because of its sodium salt content, is considered to be an absorptive dressing that creates the adequate conditions for the healing of moist wounds [19]. The NaCMC dressings that can be currently found in the market do not contain any active substances, with the exception of the silver that is added as an antimicrobial agent. A recent study by Vinklárková et al. [19] explored the incorporation of ibuprofen at a concentration of 0.5−1 mg/cm^2^ by solvent-casting into NaCMC films for buccal drug delivery. However, to the best of our knowledge, no studies on the impregnation of natural agents into NaCMC by supercritical techniques have been conducted to date.

In fact, the number of studies where the impregnation of different polymeric materials by supercritical impregnation are compared are still scarce. For instance, the SSI of NaCMC natural-based wound dressing was compared against polyester; a synthetic polymer. This polymer is considered a safe alternative to be used for the treatment of surgery wounds, since it has demonstrated to reduce early wound healing complications after arthroplasty surgery in comparison to the conventional silver-impregnated occlusive dressings [20].

Bearing in mind the potential of *Annona* plants, this study intends to determine, for the first time, the recovery by enhanced solvent extraction (ESE) of the bioactive compounds in eight different *Annona* species and three cultivars of *A. cherimola* were evaluated. The extract with the highest antioxidant potential will be later on incorporated into a synthetic (polyester dressing, PD) as well as into a natural-based wound dressing (hydrocolloid sodium carboxymethyl cellulose, NaCMC) in order to develop functionalized transdermal patches for biomedical application.

## 2. Materials and Methods

### 2.1. Raw Materials and Chemical Reagents

**Figure 1:**
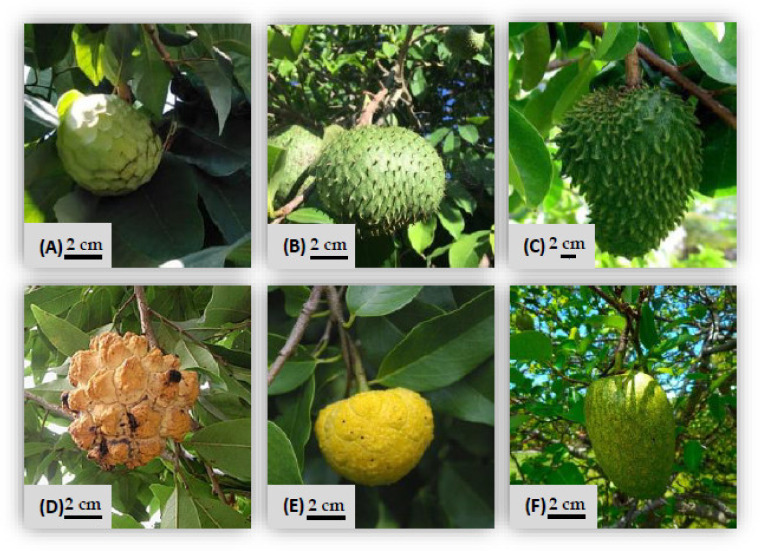
Sample images of the different *Annona* species used in this study: (**A**) *A. cherimola* (cherimoya); (**B**) *A. montana* (soursoup); (**C**) *A. muricata* (guanabana or soursop); (**D**) *A. neosalicifolia*; (**E**) *A. emarginata*; (**F**) *A. glabra* (pond apple).

The leaves from different *Annona* species (*A. cherimola* cv. ‘Fino de Jete’, ‘Alborán’ and ‘Campas’, *A. montana*, *A. muricata*, *A. neosalicifolia*, *A. emarginata* and *A. glabra*) were donated by The Subtropical and Mediterranean Horticulture Institute “La Mayora” (IHSM La Mayora–CSIC-UMA) in Algarrobo, Malaga, Spain. This research center is located in one of the major cherimoya cultivation areas, and has the most important germplasm bank of the genus *Annona* in the world. The leaves were dried at 40 °C for 24 h, ground to ~3 mm particle size and stored away from the light under moisture-controlled conditions. Sample images of the different species used in this study are shown in Figure 1.

The synthetic polyester dressing (PD) and the natural-based hydrocolloid sodium carboxymethyl cellulose (NaCMC) wound dressing (Convatec, Barcelona, Spain) were used as matrices for the impregnation experiments. The carbon dioxide (99.99% purity) was purchased from Abello-Linde S.A. (Barcelona, Spain). The organic solvents ethanol, acetonitrile, and the formic acid, all of them HPLC grade, were supplied by Panreac (Barcelona, Spain). The antioxidant capacity experiments were carried out using a 2,2-diphenyl-1-picrylhydrazyl (DPPH) reagent. For the antimicrobial assays, LB medium was prepared using tryptone, sodium chloride and yeast extract, and 2,3,5-triphenyl-tetrazolium chloride reagent (TTC) was used as the redox indicator of the viable cells. All the reagents were supplied by Sigma-Aldrich (Steinheim, Germany). The pathogenic bacteria *Escherichia coli* (CECT101) was obtained from Spanish Type Culture Collection (CECT, Valencia, Spain), and the *Staphylococcus aureus* (ATCC 6538) bacteria were purchased from Microbiologics Inc. (Saint Cloud, MN, USA). The water used in all the experiments was double-distilled milli-Q grade.

### 2.2. Enhanced Solvent Extraction of Annona Leaves

**Figure 2:**
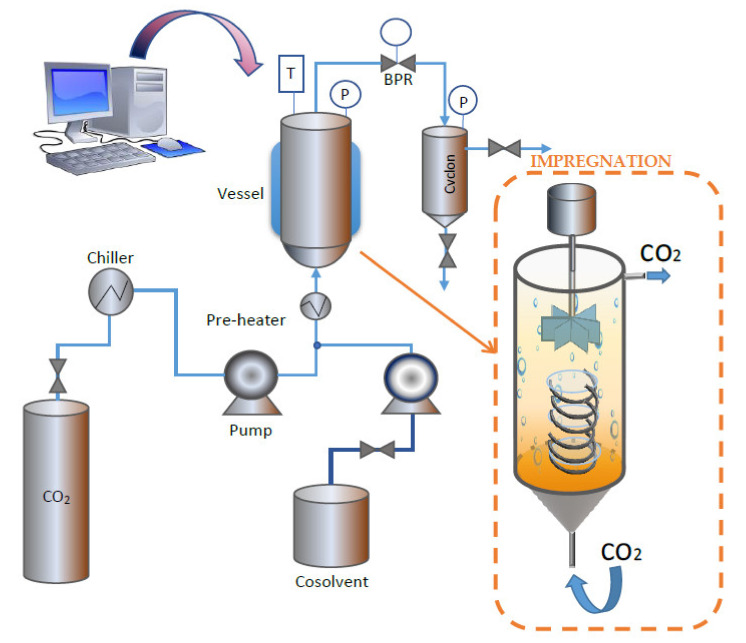
High-pressure equipment used for the extraction and impregnation processes.

The extractions were carried out in a supercritical extraction plant fitted with a 100 mL vessel (model SF100 Thar Technology, Pittsburgh, PA, USA). A simplified diagram of the equipment can be seen in Figure 2.

The six *Annona* species and the three cultivars of *A. cherimola* were evaluated: *A. cherimola* cv. ‘Fino de Jete’, ‘Alborán’ and ‘Campas’, *A. montana, A. muricata, A. neosalicifolia, A. emarginata* and *A. glabra*. For the extraction tests, ~20 g of dried *Annona* leaves in paper sachets, were placed inside the vessel. The extraction tests were carried out at constant pressure (100 bar) and temperature (80 °C). A constant 10 g/min solvent flow was applied for 2 h. The ideal extractions conditions had been established according to previous works on ESE [11]. In order to determine the polarity of the compounds present in the *Annona* leaves, two extraction solvent systems, both considered as enhanced solvents, were evaluated as follows: CO_2_ + 50% ethanol and CO_2_ + 50% acetone. The global extraction yield was determined according to Equation (1).

(1) $$\%\;Extraction\;yield=\frac{m_{e}\;\left(g\right)}{m_{AL}\;\left(g\right)}\times 100,$$

where *m_e_* is the mass of the dried extract and *m_AL_* is the initial mass of *Annona* leaves.

### 2.3. Supercritical Solvent Impregnation of the Polymeric Dressings

The impregnation experiments were carried out using a Thar Technology High-Pressure unit fitted with a 100 mL thermostated stainless cell, a P50 CO_2_ double piston pump and a Back Pressure Regulator (BPR) (Pittsburgh, PA, USA, model SF100). This equipment was modified by adding a stirring system to the impregnation vessel in order to homogenize the sample as much as possible. A simplified diagram of the equipment is shown in Figure 2. For the impregnation tests, 5 mL of *Annona* Leaf Extract (ALE) were placed at the bottom of the cell. Then, several ~300 g samples of the polymeric dressing were placed over them by means of a helical-shaped metal support, as shown in Figure 2. The support was intended to keep the dressing into place while avoiding direct contact with the ALE. A 10 g/min CO_2_ flow was applied until the desired pressure was achieved. The impregnation time was measured from that moment. The impregnations were carried out in batch mode for 1 h. An additional drying step consisting of a 5 g/min CO_2_ flow for 30 min through the cell to remove the organic solvent from the matrix was required. After that, a fast depressurization of the cell was carried out at a rate of 100 bar/min. The depressurizing rate was established based on previous studies, according to which fast depressurization rates would enhance impregnation yields [21].

Two polymeric matrices were evaluated: hydrocolloid sodium carboxymethyl cellulose (NaCMC) and polyester dressings (PD). The effects from the different impregnation conditions, including pressure (200−400 bar), temperature (35−55 °C), percentage of ethanol as co-solvent (5−10%) and the ratio active/polymeric dressing (2.6−5.3 mg ALE/100 mg polymer) were evaluated. Effectiveness was rated with respect to impregnation yields and antioxidant loadings. The antimicrobial capacity of the impregnated wound dressing was also determined.

### 2.4. Determining the Impregnation Yields

The recovering of the compounds impregnated into the polymeric matrices was based on the method described by Cejudo et al. [22]. Thus, the samples were sonicated for 30 min in 20 mL ethanol at room temperature. Then, the ethanol was removed using a rotavapor and the mass of the dried extract was measured by means of an analytical balance. The impregnation yields were then determined according to Equation (2).

(2) $$Y\;=\frac{M_{e}}{M_{i}}\times 100,$$

where Y is the impregnation yield expressed as ALE mg/100 mg dressing, *M_e_* is the mass of the dried extract recovered from the impregnated dressing and *M_i_* is the initial mass of the polymeric matrix.

### 2.5. Bioactivity of the Extracts and of the Impregnated Dressings

#### 2.5.1. Determining the Antioxidant Activity by DPPH Assays

Extracts: the antioxidant activity of the extracts was determined by DDPH assay, following the methods developed by Brand-Williams [23] and Scherer and Godoy [24]. The DPPH assays determined the antioxidant activity of the extract as the correlation between the samples’ change of color with the reduction of the 2,2-diphenyl-1-picrylhydracil (DPPH) free radical resulting from its reaction with the bioactive compounds in the samples. The absorbance decrease was determined by spectrophotometry at 515 nm. Each sample was diluted at 30–2000 µg/mL in ethanol. Then, 0.1 mL from each sample was added into 3.9 mL of 6 × 10^−5^ M DPPH. After 2 h of incubation at room temperature in the dark, the absorbance was measured at 515 nm against blank of ethanol using a visible spectrophotometer. By comparing the initial DPPH absorbance (A_0_) against the 2 h absorbance measured (A_i_) the percentage of inhibition (%I) could be determined (Equation (3)).

(3) $$\%I\;=\left(A_{0}-{\;A}_{i}\right)/A_{0}\times 100,$$

where %I is the percentage of inhibition, A_0_ is the initial absorbance at 515 nm and A_i_ is the final absorbance at 515 nm. The efficient concentration (EC_50_), defined as the concentration of extract that reduces DPPH by 50%, was graphically calculated when plotting the %I against the concentration of the extract.

The assays were carried out by duplicate and results were expressed as Antioxidant Activity Index (AAI), according to the Equation 4, where *C_DPPH_* is the final concentration of DPPH.

(4) $$\mathrm{AAI}\;=\frac{C_{\mathrm{DPPH}}}{{\mathrm{EC}}_{50}}$$

Impregnated samples: the antioxidant assay was modified according to the method described by Cejudo et al. [25]. A known weight of impregnated dressing was submerged into a 4 mL of 6 × 10^−5^ methanolic DPPH solution. The absorbance drop was monitored for 3 h at 515 nm. %I was calculated according to Equation (3), and the amount of antioxidant compounds impregnated into the wound dressing was determined by comparing the curve of %I vs. the ALE concentration previously calculated for the crude extract. The results were expressed as mg of antioxidants per g of sample (mg AOX/g). The data were calculated by triplicate.

#### 2.5.2. Antimicrobial Activity

Extracts: the extract with the highest antioxidant activity was characterized in terms of its antimicrobial activity against two pathogenic bacteria *E. coli* and *S. aureus* representative of wound infection causatives agents. *S. aureus*, an aerobic streptococcus, is the most common pathogenic isolated from wound infections leading to chronic and difficult-to-treat infections. *E. coli* is also frequently isolated from skin and soft tissue infections [26].

The assays were carried out using the reagent 2,3,5-triphenyl-tetrazolium chloride reagent (TTC), which is a redox indicator used to detect metabolic activity. In the presence of viable cells, the reagent changes the color of the medium from white into red. This color change can be measured at 490 nm. Then, by establishing a correlation between the absorbance and the bacteria concentration, the growth inhibition caused by the extract can be determined by comparison against the control samples. The analyses were carried out using 96-well microtiter plates and according to the methods described by Gabrielson et al. [27] and Moussa et al. [28] with some modifications. Each well contained 100 µL of 10^6^ ufc/mL bacteria in LB medium and 10 µL of 10 serial dilutions of ALE in a range of 500−40,000 ppm. The control samples consisted of 100 µL of 10^6^ ufc/mL bacteria and 10 µL of ethanol instead of extract. After the incubation period (24 h) at 37 °C, 10 µL of 5 mg/mL TC reagent was placed into each well. The solutions were incubated for 4 h to allow the reaction between the TTC and the growth medium to take place. Then, an Epoch 2 spectrophotometer with a microplate reader (Biotek, Winooski, VT, USA) at a wavelength of 625 nm was used for the measurements. The analyses were conducted in duplicate. The results were expressed as minimal inhibitory concentration (MIC).

Impregnated samples: the antimicrobial capacity was measured according to the method described by Cejudo et al. [29] using the bacterial strains *E. coli* and *S. aureus*. The impregnated polymer samples, previously sterilized by exposure to U.V. light for 15 min, were placed in Pyrex glass tubes (15 × 100 mm) together with 10 mL of LB medium. The tubes were incubated at 37 °C for 24 h to allow the diffusion of the compounds into the medium. After the incubation time, the absorbance at 625 nm was measured and registered as the initial absorbance (A_0_). After the diffusion step, 70 μL of inoculum adjusted to the 0.5 McFarland standard was taken, so that a concentration of 1.5∙10^6^ CFU/mL was obtained in each tube. After the inoculation, the tubes were incubated for 24 h at 37 °C. After that time, the absorbance of all the tubes were measured at 625 nm, which was considered as the final absorbance (A_i_). All the assays were carried out in duplicate, and a tube with LB medium and non-impregnated plastic was used as the control sample. Finally, the percentage of inhibition (%I) was determined for the amount of sample analyzed by applying Equation (5).

(5) $$\%I\;=\left(1-\frac{A_{i}-{\;A}_{0}}{A_{\mathrm{control}}}\right)\cdot 100$$

#### 2.5.3. Quantification of Total Polyphenols

The total phenolic content (TPC) of the extracts obtained from the different Annona species studied was determined based on the 96-well microplate Folin–Ciocalteu method given by Margraf et al. (2015) with some modifications [30]. Firstly, extract samples were diluted appropriately with MiliQ water at concentrations of 2000 ppm for ethanolic extracts and 4000 ppm for acetonic extracts. A 12.5 µL aliquot of the diluted extract was mixed with 12.5 µL Folin-Ciocalteu reagent and 200 µL MiliQ water and shaken for 1 min in a flat-bottom 96-well microplate. The mixture was left for 2 h and then 25 µL of sodium carbonate solution were added and the mixture was shaken at medium-continuous speed for 1 min. After 30 min at room temperature, the absorbance was measured at 750 nm using a microplate reader (Epoch, BioteK, Winooski, VT, USA). Gallic acid dilutions (15–300 µg/mL) were used as standards for calibration. The absorbance of the same reaction with water instead of the extract or standard was subtracted from the absorbance of the reaction with the sample. The total phenolic content was expressed as µg of gallic acid equivalents per µg dried extract (µg GA-eq/µg dried extract).

An identification of major phenolic compounds was carried out by high performance liquid chromatography (HPLC) in an Agilent Technologies Series 1100 system (Agilent, Germany) coupled to an UV/vis detector. A Synergi Hydro-RP C18 column (150 × 3 mm, internal diameter (i.d.) of 4 μm) (Phenomenex, CA, USA) with a 4.0 × 2.0 mm, i.d. C18 ODS pre-column was used. The mobile phase consisted of 0.1% formic acid prepared in water (solvent A) and 0.1% formic acid prepared in acetonitrile (solvent B) and it was eluted at a flow rate of 0.6 mL/min. The injection volume was 20 μL. The gradient profile was defined taking into consideration a previous method for phenolic compounds determination [31]. The major compounds were identified by comparison of their retention times with standard compounds and by comparing the elution behavior with that in previous studies. Additionally, the peaks identified were quantified as concentration of gallic acid in the extract (Equation (6)).

(6) $$A_{\mathrm{gallic}\;\mathrm{acid}}=411.11+75.056C$$

### 2.6. Scanning Electron Microscopy (SEM)

In order to determine the structural damages experienced by the matrices before the impregnation process and/or to detect the visible ALE particles on the impregnated dressing’s surface, impregnated and non-impregnated dressings were visually evaluated by SEM. The samples had been previously attached to a metal holder and coated with a thin layer of gold (~15 nm thick) to improve its conductivity. The analysis was conducted by means of a Quanta 200 scanning electron microscope (Thermo Fischer Scientific, Hillsboro, OR, USA) applying a voltage of 20 kV under vacuum conditions.

## 3. Results and Discussion

### 3.1. Evaluation of the Different Annona Leaf Extract Production Methods

**Figure 3:**
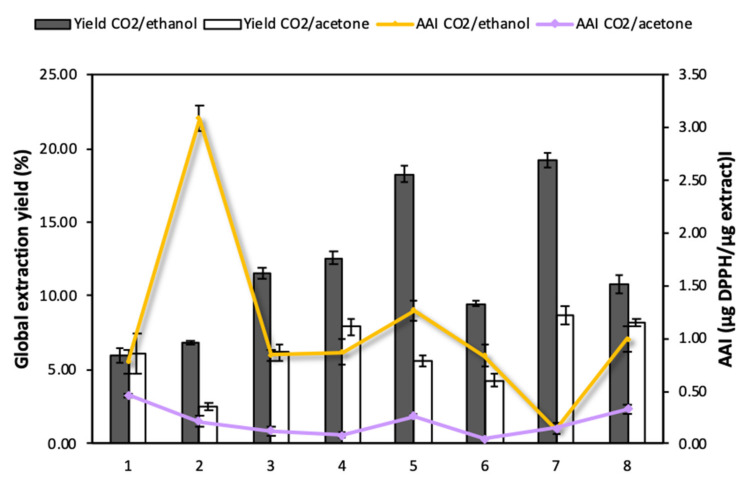
Global extraction yields and antioxidant capacity of Annona leaf extracts: 1. *A. cherimola* cv. ‘Fino de Jete’, 2. *A. cherimola cv*. ‘Alborán’, 3. *A. cherimola* cv. ‘Campas’, 4. *A. montana*, 5. *A. muricata*, 6. *A. neosalicifolia*, 7. *A. emarginata*, 8. *A. glabra*.

Enhanced solvent extraction (ESE) is renowned as an efficient technique to recover different types of bioactive compounds such as polyphenols, flavonoids, alkaloids and tannins from *Annona* bio-products [10]. It provides certain advantages in comparison to other conventional extraction methods, as it reduces the consumption of organic solvents and evaporation steps, avoids the degradation of active substances by adding high proportions of CO_2_, and enhances matter transfer phenomena. Previous studies had reported that the use of this technology increased the extraction yields of phenolic substances from leaves and skin of *A. cherimola* and obtained similar recoveries to those attained by ultrasound extraction procedures [10]. The present work aimed to determine the efficiency of ESE when applied to different *Annona* species and cultivars. The influence of the type of co-solvent (ethanol and acetone) on the process was also evaluated. Figure 3 shows the results obtained regarding overall yields (Y) and functional activity of the extracts.

#### 3.1.1. Global Extraction Yields

All of the extraction tests on the different *Annona* species analyzed were carried out at a constant pressure of 200 bar and a temperature of 80 °C. Galarce-Bustos et al. [10] reported that the use of a relatively high temperature (75 °C) favored the extraction of alkaloids and phenolic compounds from *A. cherimola* leaves and peel. On the other hand, pressures between 100 and 200 bar have been reported to be suitable for the extraction of bioactive compounds from different agro-industrial wastes [10,11,25]. Two solvent extraction systems, CO_2_ + 50% ethanol and CO_2_ + 50% acetone, were tested in this study in order to determine the best polarity conditions for the recovery of antioxidant compounds from *Annona* leaves.

The CO_2_/ethanol mixture (Y ~6.01–19.22%) proved to be more efficient for the recovery of active compounds from *Annona* leaves compared to the CO_2_/acetone mixture (Y ~2.50–8.76%) (Figure 3). This seems to indicate that the compounds that are present in *Annona* leaves have a polar character and, therefore, are more easily recovered using the CO_2_/ethanol mixture. When analyzing the specific extracts obtained from each of the *Annona* species with CO_2_/ethanol, those obtained from *A. muricata* (18.27 ± 0.54%) and *A. emarginata* (19.22 ± 0.51%) presented high extraction yields with a notable difference with respect to those obtained from the other *Annona* species. On the other hand, the samples with the lowest CO_2_/ethanol extraction yields were those corresponding to *A. cherimola* cv. ‘Fino de Jete’ and ‘Alborán’. Nevertheless, the high antioxidant capacity of the variety *A. cherimola* cv. ‘Alborán’ stands out from the others and, therefore, should be considered as the object of study for further impregnation research works.

The results in Figure 3 agree with the yields obtained using conventional extraction methods. Previous studies on the extraction of compounds from *A. squamosa* leaves by maceration in different solvents such as water, chloroform and methanol reported 8.7%, 10.1%, and 12.6% yields, respectively. Methanol proved to be more efficient than the other solvents for the extraction of flavonoids, polyphenols, alkaloids, tannins and saponins [32]. High contents of flavonoids and phenolic compounds have also been found in ethanolic extracts of *A. cherimola* [33] and *A. muricata* [34]. These authors tested different solvent mixtures and concluded that the mixture ethanol/water 80/20 (*v*/*v*) provides better results than the mixture acetone/water 70/30 (*v*/*v*). This indicates that *Annona* leaves have a high content of medium to high polarity substances, which confirms that the CO_2_/ethanol mixture is perfectly suitable for the extraction of bioactive compounds from this by-product.

#### 3.1.2. Antioxidant Activity of the Annona Leaf Extracts (ALE)

The extracts obtained using CO_2_/ethanol and CO_2_/acetone were characterized with respect to their antioxidant activity expressed in terms of Antioxidant Activity Index (AAI), where a higher value of this index would indicate a higher antioxidant activity. According to Figure 3, CO_2_/ethanol extracts (0.14–2.88 µg DPPH/µg extract) exhibited higher activity than CO_2_/acetone extracts (0.12–0.46 µg DPPH/µg extract). According to Scherer and Godoy’s scale, extracts can be classified according to AAI as follows: poor antioxidant activity when AAI < 0.5, moderate activity when 0.5 < AAI < 1, strong when 1 < AAI < 2, and potent when AAI > 2 [24]. According to this scale, all the CO_2_/acetone extracts have a poor antioxidant activity, while CO_2_/ethanol extracts from *A. cherimola cv.* ‘Alborán’, ‘Fino de Jete’ and ‘Campas’, *A. montana*, *A. muricata*, *A. neosalicifolia*, and *A. glabra* presented an antioxidant activity between “moderate” and “potent”. Among all the extracts, the one obtained using CO_2_/ethanol from *A. cherimola cv.* ‘Alborán’ stands out in particular. Even though it exhibited low yields, it is the only variety with a potent antioxidant activity level (AAI = 3.1), which is a much higher level than those reached by the other species. It was followed by *A. muricata*, whose extract showed an AAI = 1.27, corresponding to a strong activity.

It seems clear that by increasing the polarity of the solvent system with the addition of ethanol, not only the overall extraction yield improved, but also the recovery of compounds with a higher antioxidant capacity. The increase of the antioxidant capacity of *A. cherimola* cv. ‘Alborán’ leaf extract (13-fold higher) when using the mixture CO_2_/ethanol is worth mentioning. Nevertheless, this effect was not observed with other species such as *A. cherimola* cv. ‘Fino de Jete’ which exhibited a slight increase in its antioxidant capacity with the addition of ethanol as co-solvent (0.46–0.72 µg DPPH/µg extract), or *A. emerganita* which had the highest yield, but presented extracts with very low antioxidant capacity (~0.15 µg DPPH/µg extract).

Previous studies have reported that the antioxidant activity of *Annona* pulp and by-products extracts is based on their ability to mitigate free radical damage, to chelate and reduce metals, and to quench deleterious hydroxyl radicals. The extracts obtained by maceration from *A. squamosa* leaves have reported EC50 values in the range 308.3–439.6 µg/mL by DPPH assay [32]. Methanolic fractions of *A. muricata* leaves (EC50 = 10.1 µg/mL) showed not only a superior ability to quench the DPPH radical to that exhibited by ascorbic acid (EC50 = 32.6 µg/mL), but also the ability to reduce iron [35]. These data from previous studies are not comparable to those obtained in the present study, since the methods used for the DPPH assay are different. Nevertheless, they provide a reference regarding the activity values reached by extracts from other agro-industrial residues such as olive (AAI ~1.0) [22] and mango leaves (AAI = 3.3) [36]. These studies also employed CO_2_ + 50% ethanol/methanol as the solvent system and a similar protocol for the DPPH assay. The antioxidant capacity of *A. cherimola* ‘Alborán’ presented values close to those of mango leaf extracts, which is renowned for its potent antioxidant activity. This indicates the high potential of *Annona* leaf extracts regarding the recovery of this type of substance.

#### 3.1.3. Total Phenolic Content of Annona Leaf Extracts

Table 1 provides the values of the total polyphenol content (TPC) of the extracts obtained from different *Annona* species by using CO_2_/ethanol and CO_2_/acetone. Extracts obtained with CO_2_/ethanol show higher TPC values than those obtained using acetone as co-solvent. This is in correspondence with the higher antioxidant activity obtained with ethanolic extracts for the different *Annona* species. Previous studies had also revealed that the ethanolic extracts of *A. muricata* have a rather high content of phenolic compounds [34].

When correlating TPC with antioxidant activity, it can be observed that the varieties with the highest TPC, such as *A. montana* or *A. neosalicifolia*, also have high AAI values. Whereas in the case of *A. emarginata*, the low TPC content is consistent with a low AAI (Figure 3). In contrast, the varieties *A. cherimola cv. ‘*Campas’ and *A. glabra* present low TPC values but high AAI values. This effect is attributed to the presence of other substances with bioactive properties in the extracts, such as alkaloids, flavonoids or tannins. An identification of major compounds of the extract obtained by using CO_2_/ethanol from *A. muricata* is collected in Table 2. The presence of quercetin-3-glucoside, chlorogenic acid catechin and rutin are representative in the extract, which is in agreement with Cercato et al. [37].

The antioxidant activity of the extracts obtained from *Annona* by-products has been attributed to different families of antioxidant compounds. Previous studies have revealed significant correlations (*p* < 0.05) between the antioxidant status and some of the phytochemicals present in *Annona* extracts, such as polyphenols, flavonoids and alkaloids [10,36]. High contents of these compounds have been identified in the leaves, skin and pulp of species such as *A. cherimola* and *A. muricata*.

Previous studies have also revealed that ethanol is more adequate than acetone for extracting phenolic compounds from *Annona* genus plants. According to Diaz de Cerio et al. (2018) [33], flavonoids derivatives were between 63 and 76% of the total polyphenols of *A. cherimola* leaf extracts with a mixture of ethanol/water 80/20 (*v*/*v*). These authors tested different solvent mixtures and concluded that the mixture ethanol/water 80/20 (*v*/*v*) shows better results than the mixture acetone/water 70/30 (*v*/*v*). These results indicate that polyphenols present in cherimola leaves have a high polarity, which supports the use of the CO_2_/ethanol mixture in relation to the CO_2_/acetone mixture for this type of raw material.

It seems clear that *A. muricata* extract, with the highest antioxidant activity and extraction yield, was an optimal candidate for further extraction and dressing impregnation trials. Nevertheless, *A. cherimola cv.* ‘Alborán’, which, despite having a low yield, presented a potent antioxidant activity was also of obvious interest. Consequently, for comparison purposes, it was decided to carry out the impregnation tests on both varieties.

#### 3.1.4. Antimicrobial Activity of Annona Leaf Extracts

The antimicrobial activities of the selected extracts (*A. muricata* and *A. cherimola* ‘Alborán’) were tested against the pathogenic bacteria *E. coli* and *S. Aureus*. The minimal inhibitory concentrations (MIC) exhibited by the *Annona* leaf extracts are presented in Table 3.

Table 3 shows that *Annona* leaf extracts exhibit antibacterial activity against both Gram-negative and Gram-positive bacteria, but their activity was superior against the Gram-positive bacterium *S. aureus* (MIC0 < 800 µg/mL). *E. coli*, which is a Gram-negative bacterium, is more resistant to antimicrobial and antibiotic substances, as it possesses a double lipid membrane that is hard to penetrate or destroy by antimicrobial agents.

When the antimicrobial activity of the extracts was compared, it was observed that the one obtained from *A. muricata* leaves, despite having a lower antioxidant activity than that obtained from *A. cherimola* ‘Alborán’, had the capacity to inhibit bacteria, and particularly *E. coli,* even when used at lower concentrations (MIC = 1000 µg/mL). De Pinto et al. [38] also reported the effectiveness of *A. muricata* leaf extract against different Gram-negative and Gram-positive bacteria, and its antimicrobial activity was also organism dependent. In their study it is suggested that the inhibition of bacterial growth and the induction of cell death could be related to bacterial membrane destabilization. Previous studies had reported MIC values at 156 µg/mL for *S. aureus* and at 625 µg/mL for *E. coli* corresponding to methanolic extracts of *A. muricata* obtained by maceration [38]. However, such data are not comparable against those of the present study, since a different method of microbial activity was applied.

### 3.2. Evaluation of Variables in the Impregnation of ALE into the Dressings

As above mentioned, the *Annona* Leaf Extract (ALE) impregnation into wound dressings was carried out using the extracts obtained from the species *A. muricata*. Nevertheless, the high AAI exhibited by *A. cherimola* cv. ‘Alborán’ leaves extract also justifies the evaluation of this variety with respect to dressing impregnation. The impregnation of synthetic polyester dressing (PD) and the natural-based hydrocolloid sodium carboxymethyl cellulose (NaCMC) wound dressings were evaluated. The effect of the operation conditions, including pressure (*P*) within the range 200−300 bar, temperature (*T*) at 35 or 55 °C, percent of co-solvent (ethanol) at 5 or 10% ethanol concentration and ALE/polymer mass ratio between 2.6 and 5.3 mg ALE/mg polymer, were determined with regard to impregnation loading and to the functional activity of the impregnated samples. Both values, i.e., impregnation yield (*Y*) and antioxidant loading (*AL*) are presented in Table 4.

#### 3.2.1. Temperature Influence

The effect of temperature, at isobaric conditions, on both values, total impregnation yields and antioxidant compounds loadings, was determined (Table 4). On supercritical impregnation, higher temperatures diminish CO_2_ density, which makes the active compounds present a higher affinity with the matrix than with the supercritical phase. This, in turn, results in an increment of the impregnation yields. Higher temperatures also enhance diffusivity, which favors the phenomena of matter transfer into the polymeric phase.

Although impregnation yields increase drastically with increasing temperatures, no significant changes in the antioxidant loadings were observed. At 200 bar, the antioxidant loadings were similar for the two temperatures studied (35 and 55 °C), while at 300 bar just a slight increase in the loading of antioxidant compounds could be observed when the temperature was raised. The way that the extract substances, either antioxidant or non-antioxidants, compete during the impregnation process change as the operating conditions are also modified. Such competition can be both for solubility in the supercritical phase as well as to reach the active sites in the matrix. Hence, at the lowest temperature (35 °C), the impregnation of antioxidant substances was favored, even if the impregnation yields were not so high. However, when the temperature was raised up to 55 °C, not only a larger number of antioxidant substances were impregnated into the dressing matrix, but also other non-antioxidant substances, which explains greater overall impregnation yields.

#### 3.2.2. Pressure Influence

Regarding the effect of pressure on the impregnation process of ALE into the wound dressings, a pressure rise while temperature remains constant at 55 °C resulted in a significant increment of both, the impregnation yields and the antioxidant loadings (Table 4). The positive effect achieved by higher pressures on the impregnation processes has also been reported by previous studies. Thus, Dias et al. [18] and Fernández-Ponce et al. [21] reported that the impregnation loadings of gallic acid into cotton increased significantly as the temperature was increased from 100 up to 300 bar. A higher pressure improved both the solvent capability of the CO_2_ as well as the mass transfer phenomena (viscosity and diffusivity), which enhanced the solubilization of the active compounds into the CO_2_ phase and its subsequent diffusion into the matrix [21].

However, when the impregnation was performed at the lower temperature of 35 °C, the positive effect from pressure was only observed with respect to antioxidant loadings. Impregnation yields, on the contrary, seemed to be negatively affected. Thus, the corresponding density increment promoted a greater affinity of some compounds with the CO_2_ phase, which favored an easy desorption of the target compounds during the depressurization step with the subsequent lower impregnation yields [36]. Sanchez-Sanchez et al. [36] also observed a negative effect of a higher pressure on the impregnation efficiency of mango leaf extracts into polyester dressings, particularly at low temperatures.

As supercritical impregnation is a complex process, different authors have considered the relevance of the partition coefficient as a determinant factor that governs the process. The partition coefficient is defined as the ratio between the concentration of the solute in the polymer and its corresponding concentration in the CO_2_ phase. Naturally, high impregnation yields are promoted by partition coefficients that favor the affinity of the active substance with the matrix [39]. In this sense, when the extract solubility is too high, the affinity of the active compounds with the CO_2_ phase will increase, which will affect impregnation efficiency, since a smaller amount of the active substance will remain in the matrix.

**Figure 4:**
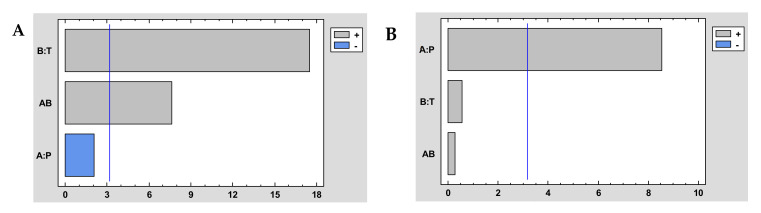
Pareto chart of impregnation yield (**A**) and antioxidant loading (**B**) of dressings at different conditions of the impregnation of *A. muricata* leaf extract into NaCMC. Tempertuare (T) and pressure (P).

It could be generally concluded that impregnation efficiency and antioxidant loadings increase with increasing pressure and temperature (Table 4). That is, a higher impregnation efficiency is achieved when operating at high pressure (300 bar) and relatively high temperature (55 °C). It was corroborated after the statistical analysis of the experiment design. The Pareto diagram of the extraction performance represented in Figure 4A shows that if the combined effect of *P* and *T* is analyzed, there is a significant increase in the impregnation efficiency when both operating variables are increased, and Figure 4B shows that the higher pressures also favored the impregnation of antioxidant compounds. Accordingly, high pressure (300 bar) and high temperature (55 °C) were applied to the subsequent experiments that intended to determine the effect of the rest of the operating conditions on the process outcome, namely ALE/polymer mass ratio and co-solvent percentage.

#### 3.2.3. Influence of ALE/Polymer Mass Ratio and Ethanol Percentage on Impregnation Efficiency

Bearing in mind that the use of high pressure and temperature has a positive effect on the impregnation process of ALE into the dressings, a pressure of 300 bar and a temperature of 55 °C were kept constant for the impregnation process of *A. cherimola* ‘Alborán’ leaves extract into polyester dressings (PD). The data obtained for different ALE/polymer mass ratios (2.6 and 5.3 mg ALE/100 mg PD) and percentages of ethanol (5 and 10%), can be seen in Table 4.

A rise of the ALE/polymer ratio in conjunction with an increment in the percentage of ethanol favors greater impregnation yields. An increase of the ALE/polymer ratio at a fixed ethanol percentage favored a higher overall yield as well as a greater amount of antioxidant substances being impregnated. However, it was necessary to increase the percentage of ethanol up to 10% to observe a more significant improvement.

This positive effect caused by the addition of a co-solvent to the supercritical phase during the impregnation process has also been reported by several studies. The effectiveness of co-solvents, such as ethanol, in the supercritical phase is associated to an enhancement of the swelling effect of scCO_2_ and to a reduction of the glass transition temperature (*T_g_*) of the polymer. scCO_2_ acts as plasticizer due to a reduction of the chain–chain interactions as well as to the lengthening of the interchain distance resulting from the swelling of the polymer [40]. It promotes the permeability of the polymer and therefore favors the penetration of the active substances. Other conventional processes add synthetic plasticizers but further cleansing steps are required if the polymer is to be used in the pharmaceutical or food industries. Therefore, the use of scCO_2_ as a reversible plasticizer is highly convenient to obtain solvent-free products [39]. In addition, the plasticizing effect of scCO_2_ takes place at low temperatures, which favors the preservation of the thermally sensitive compounds or biomaterials [40].

Despite the significant improvement in the overall performance associated to the co-solvent %, a decrease in antioxidant loadings is observed when the co-solvent percentage exceeds certain limits. In fact, a higher percentage of co-solvent in the reactor may cause a shift in the equilibrium of the system. As previously mentioned, the compounds compete for both solubilities into the supercritical phase and affinity with the matrix. Thus, an excessive increment in ethanol percentage may favor not only the solubility of the antioxidant compounds, but also that of non-antioxidant substances, which would result in a reduction of the antioxidant loadings impregnated onto the matrix (Table 4).

### 3.3. Effectiveness of the Impregnation Process of Different Polymer Matrices

A natural-based hydrocolloid sodium carboxymethyl cellulose (NaCMC) wound dressing and a synthetic polyester dressing (PD) were evaluated in this study. When both polymer dressings were compared in the present work, similar impregnation yields were obtained: ~0.40−0.82 mg/100 mg PD and ~0.12−1.47 mg/100 mg NaCMC (see Table 4). However, the supercritical impregnation with *Annona* leaf extract produced quite varied antioxidant loadings. The PD dressings incorporated 100-fold the amount of antioxidants (~10.5−15.5 mg AOX/g) impregnated into the NaCMC dressings: ~0.17−0.23 mg AOX/g (Table 4). The PD dressings that had been impregnated with *A. cherimola cv.* Alboran leaf extract, had a significantly higher antioxidant potential than those impregnated with *A. muricata* extract (Figure 3). However, the substantial difference between the antioxidant loadings in PD—100-fold greater—and NaCMC antioxidant loadings, indicates that the supercritical impregnation of ALE was also dependent on the polymeric material. This seems to indicate that the affinity of the active substance with the polymer matrix may be governed by the hydrophobic/hydrophilic nature of each particular polymer. Furthermore, the degree of crystallinity of the material and its glass transition temperature are also crucial factors to determine the swelling of the polymer in the presence of scCO_2_ [41,42].

**Figure 5:**
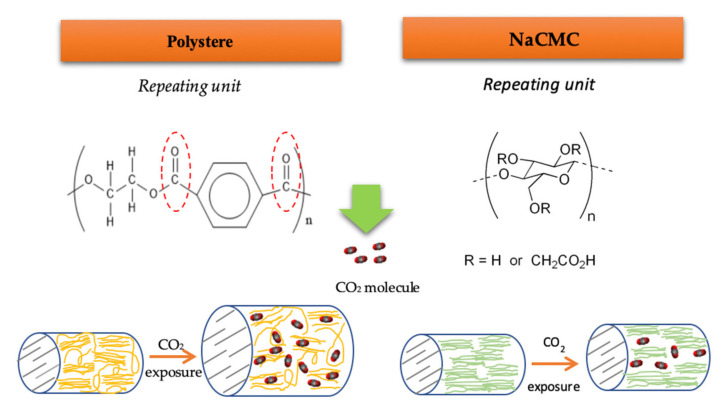
Simplified representation of the chemical structures and the swelling mechanism in PD and NaCMC dressings when subjected to scCO_2_.

In agreement with the affinity of ALE for the different polymers, it was observed that lower amounts of antioxidants were loaded into NaCMC dressings compared to those loaded into PD ones (Table 4). The monomer structure of NaCMC is shown in Figure 5, where R stands for -H or -CH_2_CO_2_Na. The degree of substitution (D.S.) is the average number of carboxymethyl groups that are replaced per monomer unit, ranging from 0 to 3 with the remaining R=H. NaCMC contains carboxyl and hydroxyl groups that may establish strong hydrogen bonds with polar active molecules. However, the crystallinity and hydrophobicity of NaCMC is accepted to be a function of its D.S. In this sense, its degree of crystallinity is lower than that of cellulose when its D.S. is increased. Whereas, a higher hydrophobicity was correlated with a lower D.S [43]. Keeping this in mind, it should be considered that the specific NaCMC dressings that were used in the present study may exhibit higher hydrophobicity and higher crystallinity because of a low D.S, and this fact would negatively affect the impregnation of antioxidant compounds.

Moreover, the swelling effect on NaCMC is restricted by the organized structure of cellulose and also by a high *T_g_* (~121 °C) [44]. Cellulose chains form intra and inter-molecular bonds, which results in an organized chain structure with a high crystallinity [21]. It is a fact that the diffusion of the solute over the crystalline regions is limited, so the scCO_2_ sorption (plasticizing effect) can only take place at the amorphous regions. Ethanol also acts as plasticizer in supercritical processes, but it can promote a swelling effect only in the amorphous region of the NaCMC.

A diagram of the swelling mechanisms of NaCMC and PD dressings is show in Figure 5. The swelling effect on the amorphous regions of NaCMC could be not enough to facilitate the diffusion of the active compounds into the matrix, which would result in poor antioxidant loadings. Nevertheless, according to the large impregnation yields attained, considerable amounts of compounds were impregnated into the NaCMC dressing, which indicates that a number of compounds other than the antioxidant compounds in the ALE had impregnated the polymer because of their higher affinity with the same.

Polyester, on the other hand, is a product composed of hydrocarbon chains of ethylene groups and terephthalate groups containing ester bonds. Polyester has a crystalline structure and a glass temperature (*T_g_*) as high as 125 °C [45]. Nevertheless, under scCO_2_ fluid, the polymer’s *T_g_* decreases and facilitates molecule vibrations. Macromolecular chains initially gain rotational freedom and begin to take up more space. This causes the polymer chains to rearrange to absorb more CO_2_ because of the increment in free volumes. Han et al. reported that the swelling effect on polyester increases with pressure and temperature [45]. This enhanced swelling behavior of PD in scCO_2_, which if further favored by the addition of ethanol, facilitates the diffusion of the ALE molecules into the polyester fibers. The antioxidant molecules in the ALE have a greater affinity with PD than with NaCMC dressings, which favors the loading of these molecules rather than the other non-antioxidant compounds that could compete for the active sites in the PD dressings. As a result, a larger amount of antioxidant compounds can be loaded into the synthetic PD dressings.

When compared to other studies on the impregnation of plant extracts into dressings, the antioxidant loadings achieved by ALE into PD dressings were quite remarkable. Previous studies on the impregnation of mango leaf extract into PD samples resulted in dressings with a lower antioxidant load than those obtained in the present study (2.8 mg AOX/100 g) [36]. The impregnation loadings of mango leaf extract into cotton fabric samples were better (~1.0 mg AG/g cotton) [21], but still poorer than those achieved in the present work into PD dressings (Figure 4B). Similar levels of impregnation loadings have been observed in other works where supercritical impregnation was applied to other natural antioxidant substances such as quercetin into agarose foams and chitosan (~25 mg/g) [39]. Our results confirm the potential of PD dressings to incorporate a high loading of ALE antioxidant substances by means of SSI.

Keeping in mind that the final purpose of the developed product is wound healing, the incorporation of an antimicrobial agent into the PD dressing would represent an additional benefit to this type of product that would contribute to prevent wound infections. For this reason, the antimicrobial activity of the dressings showing the highest antioxidant activity (those obtained using PD and impregnated with the *A. cherimola* ‘Alborán’ extract at 300 bar, 55 °C, 10% ethanol and 5.3 mg ALE/100 mg) was evaluated. Table 5 shows the microorganisms used for the test, the concentration of extract released into the medium from the impregnated dressings, and the percentage of bacterial inhibition attained.

Considering the characterization data of the crude extract with respect to its antimicrobial activity (Table 3), the inhibition percentages obtained by the dressings, 38.7% for *S. aureus* and 24.1% for *E. coli*, are in accordance with those corresponding to the extract. The %I of *S. aureus* (Gram+) is higher—a less resistant bacteria that is highly susceptible to antibacterial substances such as polyphenols—than that of *E. coli*. The results are also in accordance with the concentration levels of the extract released from the dressings into the culture medium. The inhibition percentages below 100% were calculated because the concentration of ALE in the medium did not match the MIC of the crude extract (Table 5).

### 3.4. Characterization of the Surface of the Impregnated Dressings

**Figure 6:**
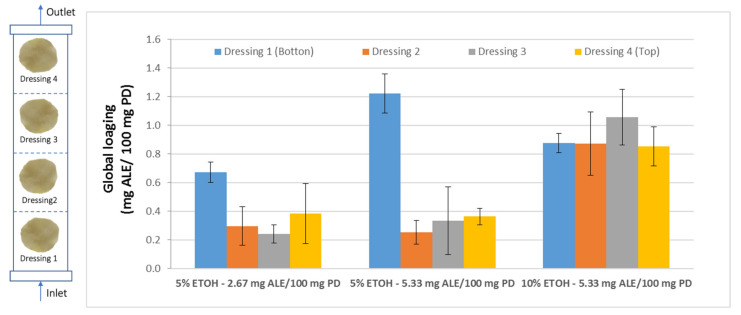
Total impregnation loadings of the samples located at four different heights inside the reactor and using three different configurations for ethanol percentage and ALE/polymer mass ratio.

Once the influence of the operating variables on the impregnation process of *Annona* leaf extract into wound dressings was determined, it was necessary to verify the suitability of the impregnated product for its potential biomedical use. This verification should not only include a study on how the solute is impregnated on the surface of the dressing, but also, in view of a possible scaling-up of the process, the impregnation capacity of the dressings should be determined as a function of their position inside the reactor during the impregnation process. This should ensure a more consistent and higher quality product. For this study, four samples were placed at different heights inside the reactor separated from each other by a metal holder (Figure 2). Then, the impregnation processes were carried out using two different percentages of ethanol (5–10%) and two different ALE/PD ratios (2.67–5.33 mg ALE/100 mg PD) at four different heights. The overall impregnation loadings of each set of samples located at four different heights inside the reactor were determined. Figure 6 shows the data corresponding to the different samples under the conditions studied.

The samples that were impregnated using 5% ethanol exhibited very similar loadings, regardless of the ALE/PD ratio, except in the case of the samples located at the bottom position, where a higher ratio would lead to a greater loading. In any case, the samples located at the bottom of the reactor presented significantly greater loadings than the rest of the samples. This is due to the fact that those samples are closer to the liquid extract at the bottom of the reactor. Although all the samples were separated from the base of the reactor by the metal holder, the bottom samples are the first ones to come into contact with the extract and the ones that are most easily impregnated by the active substances from the extract.

**Figure 7:**
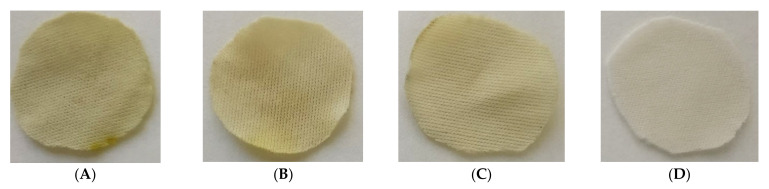
The first three images on the left are the dressings impregnated according to the following conditions: (**A**) 5% ETOH—2.5 mg ALE/100 mg PD, (**B**) 5% ETOH—5.3 mg ALE/100 mg PD and (**C**) 10% ETOH—5.3 mg ALE/100 mg PD, (**D**) is an non-impregnated sample.

However, when the percentage of ethanol is increased from 5 to 10%, not only the overall impregnation loadings grow but also a significant improvement of the uniformity of the samples can be noticed. No statistically significant differences can be observed between samples when placed at different heights within the reactor. Similar impregnation loadings were obtained in all the samples. Achieving impregnation homogeneity along the reactor is a key factor in view of a potential scale-up to industrial level, where the final products must comply with quality standards that demand consistency of the loadings as well as of the functional properties. Figure 7 shows different polyester dressings impregnated with ALE under different operating conditions.

**Figure 8:**
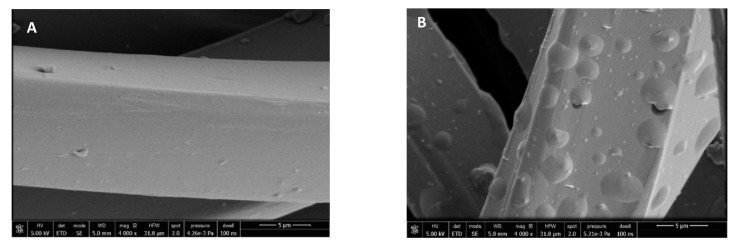

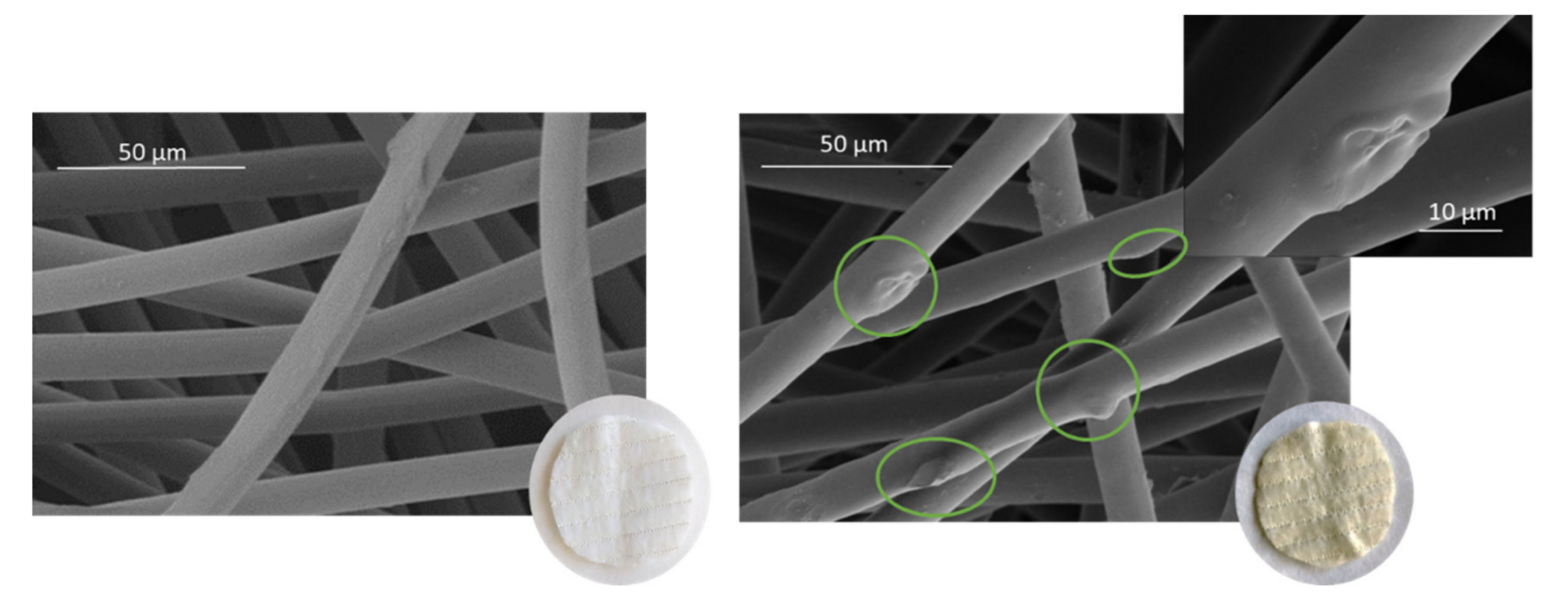
SEM images of the wound dressings before and after impregnation: PD (**A**) and NaCMC (**B**).

Figure 8 shows that the surface of the fibers is similar before and after impregnation. Therefore, it was microscopically verified that the process does not alter the structure of the polyester fibers. The small vesicles embedded into the fibers that can be seen in the images correspond to the impregnated extract particles. The vesicles that can be seen impregnated into the fabric have semi-spherical shapes and different sizes. This differs from the images corresponding to mango extract impregnation [31], where the PD-impregnated mango leaf extract particles showed a distinctive spherical shape. These differences in particle shapes may be due to the way the particles precipitate from each extract and also to the different types of interactions between the matrix and the extract compounds. The SEM images allowed us to verify that the ALE impregnation process in PD dressings was highly efficient, as the number of vesicles that could be detected in a small region of the cloth, viewed at a magnification of 4000× and 50 µm, is quite high, and even greater than that observed when using mango extract [36].

Similarly, the structural changes of the ALE-impregnated NaCMC dressings were analyzed by SEM. It was also observed that the matrix fibers were not altered. Although in this case, fewer vesicles were observed to be impregnated in the NaCMC dressings compared to the polyester ones. Only some protrusions and embeddings could be observed in the NaCMC fibers. However, no particles of distinct shapes, such as semi-spherical particles deposited on the polyester fibers, could be detected. This indicates that there are differences in the way the particles are deposited on the different polymers, both due to the active substance/polymer interactions and also possibly because of the swelling experienced by polymer.

## 4. Conclusions

The potential of leaf extracts from different *Annona* species and varieties as a source of antioxidant and antimicrobial substances was demonstrated in the present study. Varieties such as *A. cherimola* ‘Alborán’ and *A. muricata* stood out for their strong antioxidant activity compared to the other species studied, and the use of a polar system of CO_2_ + 50% ethanol was confirmed as a suitable system for the recovery of the active substances of interest. The potential of these extracts to impregnate dressings for biomedical applications was also evaluated. A number of PD and NaCMC matrices were impregnated obtaining different yields and antioxidant loadings. The application of supercritical impregnation processes at relatively high temperature (55 °C) and high pressure (300 bar) was confirmed to improve impregnation yields. Thus, on the one hand, the solubility and diffusivity of the active substances is increased, while, on the other, the high pressure applied enhances the swelling effect of scCO_2_ on the polymer. Similarly, a higher ALE/polymer ratio and a higher percentage of ethanol as the co-solvent, up to 10%, significantly improved the impregnation performance. Ethanol exhibits a plasticizing effect that favors the swelling effect on the polymer and brings down the glass transition temperature, all of this facilitates the access of the extract into the active sites of the polymer. The PD dressings exhibited a higher bioactivity probably based on the greater swelling effect of this polymer in the presence of scCO_2_ and on the affinity of its carbonyl groups with substances of a polar nature such as the antioxidant compounds in the extract. On the other hand, the NaCMC polymer dressings presented a poorer CO_2_ sorption and a lower particle diffusion due to its rigid, highly ordered and crystalline molecular structure. This resulted in a reduced access of the antioxidant substances into the active sites within the NaCMC fibers, which negatively affected impregnation efficiency. The PD dressings not only exhibited a high antioxidant capacity but also antibacterial capacity against *S. aureus* and *E. coli*, bacteria which is a factor of major importance towards potential wound contaminations. It was, therefore, confirmed that *Annona* extracts and particularly those obtained from some of its varieties (*A. cherimola* ‘Alborán’ and *A. muricata)* present a promising potential for PD based transdermal systems to be used for the healing of wounds in biomedical applications.

## Figures and Tables

**Table 1:** Total phenolic content (TPC) of leaf extracts obtained from different species of *Annona* by using CO_2_/ethanol and CO_2_/acetone in terms of µg AG/µg dried extract.

Annona Specie Extract	TPC CO_2_ + 50% Ethanol	TPC CO_2_ + 50% Acetone
1. *A. cherimola cv. ‘Fino de Jete’*	55.14 ± 1.82	38.56 ± 1.37
2. *A. cherimola cv.* *‘Alborán’*	54.90 ± 1.99	34.81 ± 0.76
3. *A. cherimola cv.* *‘Campas’*	19.98 ± 1.45	8.96 ± 1.24
4. *A. Montana*	34.61 ± 1.83	11.88 ± 0.59
5. *A. muricata*	65.17 ± 1.99	32.29 ± 0.75
6. *A. neosalicifolia*	24.07 ± 1.64	31.24 ± 1.32
7. *A. emarginata*	24.30 ± 2.82	36.66 ± 1.43
8. *A. glabra*	42.22 ± 2.15	37.97 ± 0.47

**Table 2:** Chemical composition of the ethanolic extract from *A. muricata* by HPLC (280 nm).

Phenolic Compounds	Retention Time	Phenolic Content of Extract (µg/mL Extract)
Gallic acid	4.22	7.99 ± 0.89
Chlorogenic acid	11.51	3.25 ± 0.59
I 3-C-(2-O-p-hydroxybenzoyl)-β-D-glucoside	19.93	21.65 ± 1.67
Catechin	24.79	23.12 ± 1.26
Quercetin 3-glucoside	34.99	19.65 ± 1.11
Rutin	36.37	8.15 ± 0.65
Quercetin	42.36	6.25 ± 1.02
Kaempferol	46.91	46.05 ± 2.12

**Table 3:** Antibacterial activity of the *Annona* leaves extracts obtained from *A. cherimola cv.* ‘Alborán’ and *A. muricata* using CO_2_ + 50% ethanol.

*Annona* Species/Variety	*S. aureus* MIC ^a^	*E. coli* MIC
*A. cherimola cv.* ‘Alborán’	800	2000
*A. muricata*	750	1000

^a^ Minimal inhibitory concentration expressed as µg/mL.

**Table 4:** Impregnation yield and antioxidant loading of dressings under different conditions for *A. muricata* leaf extract into NaCMC and *A. cherimola cv.* Alboran leaf extract into PD.

Wound Dressing	*Annona* Species	Impregnation Condition	Impregnation Yield (mg ALE/100 mg PD)	Antioxidant Loading (µg AOX/mg Dressing)
Sodium carboxymethyl cellulose (NaCMC)	*A. muricata*	200 bar 35 °C	0.59 ± 0.20	0.16 ± 0.05
		200 bar 55 °C	0.13 ± 0.10	0.21 ± 0.03
		400 bar 35 °C	1.07 ± 0.14	0.17 ± 0.05
		400 bar 55 °C	1.47 ± 0.18	0.23 ± 0.04
Polyester dressing (PD)	*A. cherimola* 400 bar and 55 °C	5% EtOH—2.67 mg ALE/100 mg PD	0.40 ± 0.12	12.89 ± 1.65
		5% EtOH—5.33 mg ALE/100 mg PD	0.49 ± 0.15	16.36 ± 3.62
		10% EtOH—5.33 mg ALE/100 mg PD	0.82 ± 0.11	11.11 ± 2.56

**Table 5:** Antimicrobial activity of the polyester dressings impregnated with ALE at 400 bar, 55 °C and using 10% ethanol and 5.3 mg ALE/100 mg PD ratio.

Bacterial Strain	Concentration in the Culture Media (µg ALE/mL)	% Inhibition
*S. aureus*	340	38.70
*E. coli*	500	24.10

## Data Availability

Data is contained within the article.
